# Katanin Effects on Dynamics of Cortical Microtubules and Mitotic Arrays in *Arabidopsis thaliana* Revealed by Advanced Live-Cell Imaging

**DOI:** 10.3389/fpls.2017.00866

**Published:** 2017-05-24

**Authors:** George Komis, Ivan Luptovčiak, Miroslav Ovečka, Despina Samakovli, Olga Šamajová, Jozef Šamaj

**Affiliations:** Department of Cell Biology, Faculty of Science, Centre of the Region Haná for Biotechnological and Agricultural Research, Palacký University OlomoucOlomouc, Czechia

**Keywords:** Arabidopsis, cell division, interphase, katanin, live imaging, microtubules, preprophase band, super resolution microscopy

## Abstract

Katanin is the only microtubule severing protein identified in plants so far. Previous studies have documented its role in regulating cortical microtubule organization during cell growth and morphogenesis. Although, some cell division defects are reported in *KATANIN* mutants, it is not clear whether or how katanin activity may affect microtubule dynamics in interphase cells, as well as the progression of mitosis and cytokinesis and the orientation of cell division plane (CDP). For this reason, we characterized microtubule organization and dynamics in growing and dividing cotyledon cells of Arabidopsis *ktn1-2* mutant devoid of KATANIN 1 activity. In interphase epidermal cells of *ktn1-2* cortical microtubules exhibited aberrant and largely isotropic organization, reduced bundling and showed excessive branched microtubule formation. End-wise microtubule dynamics were not much affected, although a significantly slower rate of microtubule growth was measured in the *ktn1-2* mutant where microtubule severing was completely abolished. KATANIN 1 depletion also brought about significant changes in preprophase microtubule band (PPB) organization and dynamics. In this case, many PPBs exhibited unisided organization and splayed appearance while in most cases they were broader than those of wild type cells. By recording PPB maturation, it was observed that PPBs in the mutant narrowed at a much slower pace compared to those in Col-0. The form of the mitotic spindle and the phragmoplast was not much affected in *ktn1-2*, however, the dynamics of both processes showed significant differences compared to wild type. In general, both mitosis and cytokinesis were considerably delayed in the mutant. Additionally, the mitotic spindle and the phragmoplast exhibited extensive rotational motions with the equatorial plane of the spindle being essentially uncoupled from the division plane set by the PPB. However, at the onset of its formation the phragmoplast undergoes rotational motion rectifying the expansion of the cell plate to match the original cell division plane. Conclusively, KATANIN 1 contributes to microtubule dynamics during interphase, regulates PPB formation and maturation and is involved in the positioning of the mitotic spindle and the phragmoplast.

## Introduction

The diversity of plant cell forms and sizes, largely owes to the capacity of plant cells to organize intricate patterns of cytoskeletal elements and particularly microtubules. Plant cortical microtubules control cellulose deposition during cell growth and morphogenesis (Paredez et al., 2006; Lei et al., 2015) and are also involved in environmental plant responses (Dovgalyuk et al., 2003; Nyporko et al., 2003). Moreover, mitotic microtubule arrays regulate cell division plane orientation by assembling the preprophase microtubule band (PPB; Rasmussen et al., 2011) and finally drive the partitioning of genetic material between two daughter cells following the processes of mitosis and cytokinesis.

The decision between symmetric and asymmetric division is highlighted by the respective symmetric or asymmetric PPB positioning (Rasmussen et al., 2011). PPB is formed during interphase by restructuring of the diffuse cortical microtubule system into a broad cortical microtubule ring that progressively narrows and finally disassembles at the mitosis onset coincidentally with nuclear envelope breakdown (Van Damme et al., 2007). The process of cortical microtubule rearrangement to the formation of the PPB is poorly understood, but it is consistent with mechanisms generally involved in microtubule organization including hybrid treadmilling and dynamic instability (Shaw et al., 2003; Komis et al., 2014), microtubule bundling (Hamada, 2014), and nucleation (Janski et al., 2012; Fishel and Dixit, 2013). Such processes are driven by microtubule associated proteins which bind to microtubule walls or tips and regulate microtubule dynamics, interactions, and/or microtubule positioning (Hamada, 2014).

Spindle assembly occurs shortly before the complete disassembly of the PPB and assumes its bipolarity quite early. After PPB and nuclear envelope are completely disassembled the mitotic spindle interacts with chromosome kinetochores and partitions sister chromatids to two equivalent groups. Afterwards, the two chromosome groups are physically separated by the cell plate, the daughter wall which is built centrifugally through a microtubule based machinery, the phragmoplast. It is notable that throughout mitosis and cytokinesis the equatorial plane of the spindle and the plane of cell plate expansion are very tightly related to the plane set earlier by the PPB (Rasmussen et al., 2011).

The above brief description of plant microtubule properties, reveals a very dynamic structure that undergoes large scale transitions in relatively short time. The cortical microtubule array which is responsible for cell growth directionality and differentiation, can reorganize promptly after the perception of hormonal (Soga et al., 2010) or physical (Blancaflor and Hasenstein, 1993, 1995a,b; Krasylenko et al., 2012; Uyttewaal et al., 2012; Muratov and Baulin, 2015) stimuli to promote cell growth at certain direction. Otherwise it can massively reorganize and initiate symmetric or asymmetric divisions depending on how the PPB will be arranged. In a similar line, transitions between successive mitotic stages, reveal that the mitotic spindle also undergoes dramatic form shifts, while in the end, the timely and positionally accurate cell plate deposition will depend on phragmoplast microtubule dynamics.

Extensive research over the years, revealed that such transitions are facilitated and coordinated by a number of microtubule associated proteins (Hamada, 2014) including KATANIN 1 (Nakamura, 2015 and references therein).

By contrast to mammalian cells which harbor three AAA-ATPase microtubule severing enzymes, namely, katanin (McNally and Vale, 1993), spastin (Roll-Mecak and Vale, 2005), and fidgetin (Zhang et al., 2007) plants as represented by *Arabidopsis thaliana* and rice only seem to express *KATANIN* (Nakamura, 2015). The product of *KATANIN 1* gene of Arabidopsis encodes for the catalytic p60 subunit of katanin, while the regulatory 80 kDa subunit seems to be absent, although four orthologues have been reported (Keech et al., 2010) but without any functional evidence. Even so, *in vitro* experiments showed that the p60 subunit of Arabidopsis is capable of exerting microtubule severing activity (Stoppin-Mellet et al., 2002).

By mostly studying mechanisms of microtubule reorganization in elongating hypocotyl epidermal plant cells, it was found that the severing activity of katanin favors the biased parallel arrangement of cortical microtubules by distinct mechanisms (Nakamura, 2015). First of all, KATANIN 1 severs nascent microtubules that are nucleated on the walls of preexisting ones by means of γ-tubulin and augmin mediated nucleation (Murata et al., 2005; Nakamura et al., 2010; Liu et al., 2014). KATANIN 1 severing activity is also activated at points of microtubule crossovers (Wightman and Turner, 2007) as it is often observed during environmentally inducible changes in microtubule organization (Lindeboom et al., 2013).

The roles of KATANIN 1 in the transition from interphase to mitosis with the formation of the PPB and subsequently in the dynamics of the mitotic spindle and the centrifugal expansion of the cytokinetic phragmoplast remain largely elusive as only three previous studies addressed mitotic microtubule organization exclusively in fixed root cells of three *KATANIN 1* mutants *fra2, lue1*, and *ktn1-2* using immunolocalization technique (Burk et al., 2001; Panteris et al., 2011; Panteris and Adamakis, 2012).

Herein we chose to study microtubule dynamic organization in a knockout *KATANIN 1* mutant *ktn1-2* (Nakamura et al., 2010). To circumvent disadvantages of static imaging in fixed cells, we study microtubule organization and dynamics in interphase and dividing cells of *ktn1-2* stably expressing an appropriate microtubule marker GFP-TUA6. Using both high-resolution and fast advanced microscopy platforms such as structured illumination microscopy (SIM), spinning disc, and Airyscan confocal laser scanning microscopy, we uncover novel functions of KATANIN 1 on microtubule dynamics during cell cycle.

## Materials and methods

### Plant material

*A. thaliana* wild type Columbia (Col-0) ecotype and *ktn1-2*, a T-DNA mutant were used. For germination, Col-0 and mutant seeds were surface sterilized, plated on 0.8% w/v Phytagel® solidified 1/2 Murashige and Skoog medium (1/2 MS; Duchefa) with 1% w/v sucrose, stratified for 1–4 days at 4°C and subsequently transferred to environmental chamber with controlled light/dark cycle, temperature, and humidity.

### Chemicals

Unless stated otherwise, all common chemicals were from Sigma and were of analytical grade. FM4-64 was from Invitrogen and it was used at a final concentration of 5μg/ml diluted in half-strength liquid MS medium.

### Transgenic line preparation and selection

For live cell imaging Col-0 and *ktn1-2* mutants stably expressing a GFP-TUA6 marker were used. For generating transgenic *ktn1-2* line with GFP-TUA6, *ktn1-2* homozygotes (Nakamura et al., 2010) were crossed with Col-0 plants stably transformed with a *35S::TUA6:GFP* construct (Shaw et al., 2003). For imaging purposes, 7–10 day old seedlings grown from F2 seeds were used after selection for obvious *ktn1-2* phenotype and expression of GFP.

### Microscopy

For live imaging of microtubules in the *ktn1-2* mutant we used four different Zeiss microscopy platforms (Zeiss Microscopy, Oberkochen, Germany) including an LSM710 spectral CLSM, a Cell Observer, spinning disc, an LSM880 with Airyscan and an Elyra PS.1 unit for SIM (Komis et al., 2014, 2015). For documentation of cortical microtubule dynamics we used either SIM coupled to a PCO. Edge 5.5 sCMOS camera (Komis et al., 2015) using the 488 nm line of an Argon laser for excitation and appropriate filter cube for emission, or the LSM710 with 4-line averaging and pinhole set to 1 Airy unit for GFP coupled to a 32 GaAsP detector. For documenting mitotic spindle dynamics we rather used a Cell Observer spinning disc system with a 25 mW multiline Argon laser (which was set to 50% of laser power of the 488 nm line for every experiment), coupled to an Evolve 512 EM CCD camera, setting laser power and camera exposure time (typically 100 ms) to the minimum possible to allow acquisition of 3D time series. Alternatively, we have used also LSM880 with Airyscan and single photon excitation with the 488 nm line of a multiline argon laser with the same specifications as mentioned before (25 mW for the 488 nm line) and a 32 GaAsP detector. Due to the superior light collecting capacity of the Airyscan and the sensitivity of the GaAsP detector, laser power never exceeded 1% of the laser. In this case samples were either scanned with the Fast mode to allow sufficient temporal resolution during 3D-time acquisition, or with the super-resolution mode to allow optimum 3D resolution for acquiring single Z-stacks. To avoid phototoxicity perturbations and temporal delays during the documentation of the mitotic progress, we preferably used the Airyscan CLSM and the spinning disc microscope for recording mitotic cells, since they allowed the most efficient light collection compared to other platforms and also a fast scanning mode of the sample. The ability of cells to progress through mitosis and complete cytokinesis without disturbances was considered as proof of lack of phototoxicity for the duration of each respective experiment.

### Image analysis

Images acquired with the above microscope systems were post-acquisition analyzed. Angular distribution of cortical and PPB microtubules was deciphered by means of Cytospectre (Kartasalo et al., 2015). Using this software, it is possible to decipher the angular distribution of cortical microtubules irrespectively of their polarity (since GFP-TUA6 labels the microtubule lengthwise) and thus deduce their net orientation as “transverse” (i.e., perpendicular to the cell axis) at angles of 90° or 270°, “longitudinal” (i.e., parallel to the cell axis) at angles of 0° or 180° and random at angles 0°<φ <180°. The software also provides an estimate of the ratios of polymers per angle (0–1 at 0.2 increments; concentric cycles in the respective graphs).

For quantifying fluorescence skewness (a measure of microtubule bundling) we used previously published macros (Higaki et al., 2010) which were developed for Image J (http://rsb.info.nih.gov/ij/) on SIM images of Col-0 or *ktn1-2* petiole or cotyledon epidermal cells carrying a GFP-TUA6 molecular marker for microtubules. These macros belong to the formerly known KBI suite from Hasezawa lab and can be now found in LPixel ImageJ Plugins (https://lpixel.net/products/lpixel-imagej-plugins/).

For microtubule dynamic analyses, kymographs were generated using the appropriate Kymograph plugin of Zeiss Zen Blue 2014 (Zeiss Microscopy, Oberkochen, Germany), or the MultipleKymograph plugin for Image J (https://www.embl.de/eamnet/html/kymograph.html). From such kymographs, variables such as end-wise growth and shrinkage rates, catastrophe, and rescue frequencies were calculated. In particular, growth and shrinkage rate were calculated from the growth and shrinkage slope of the respective kymograph. Catastrophe or rescue frequencies were calculated by dividing the total number of events recorded (catastrophes and rescues, respectively) by the total time spent in the growing (for catastrophes) or shrinking (for rescues) phase during which these events were counted (Verde et al., 1992) for each microtubule recorded. Severing events in Col-0 and *ktn1-2* mutants were measured from SIM videos and were expressed as events × 10^−3^/μm^2^/min.

### Statistical evaluation

Wherever applicable wild type and *ktn1-2* microtubule dynamics parameters were compared by two-tailed Student's *t-*test. Statistical significance was deemed when *p* < 0.05.

## Results

### Organization and dynamics of cortical microtubules

**Figure 1:**
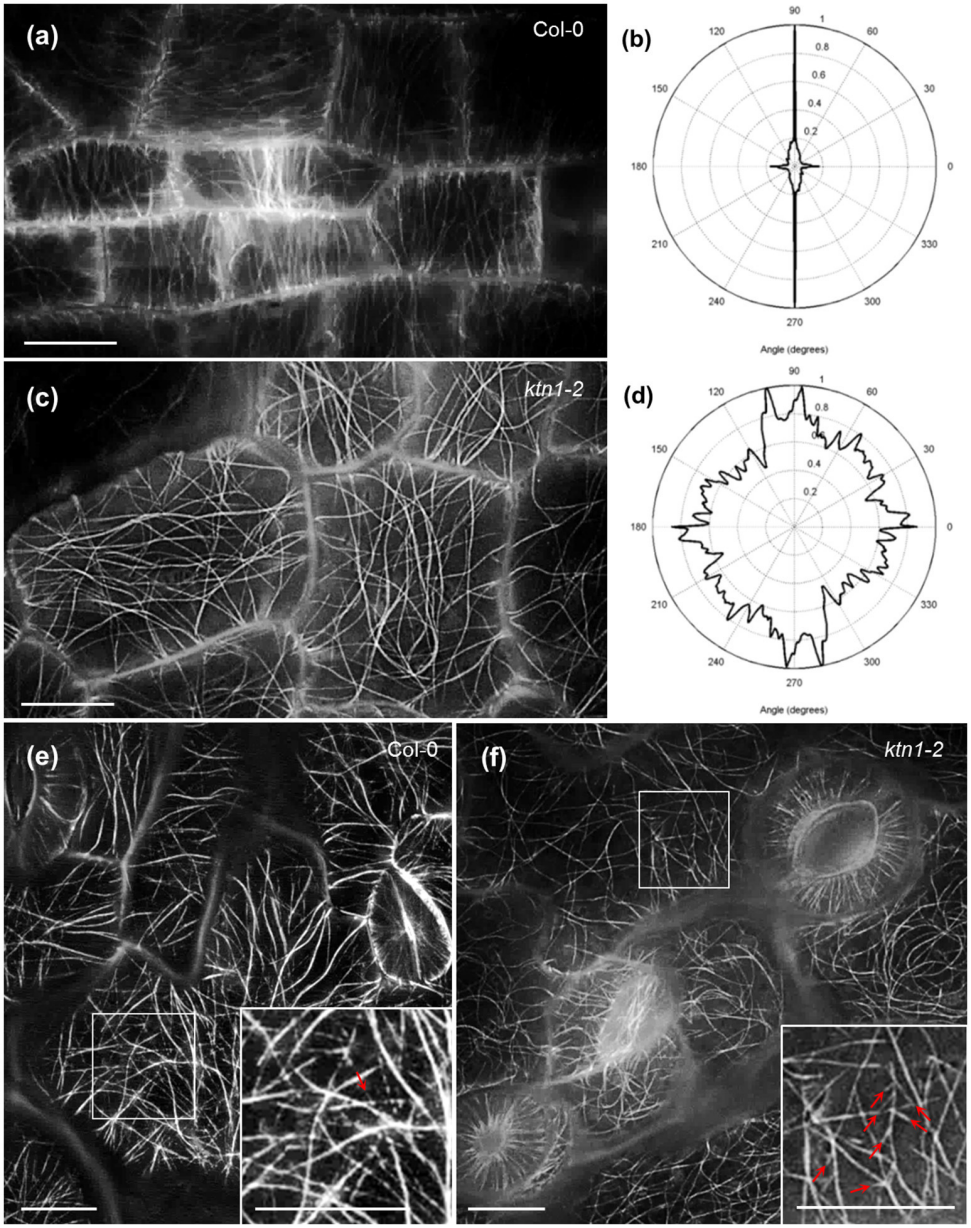
**Cortical microtubule organization in petiole and cotyledon epidermal cells of Col-0 and ***ktn1-2*** mutant expressing a GFP-TUA6 microtubule marker after SIM 2D imaging. (a,b)** Prevalent transverse orientation of cortical microtubules in Col-0 petiole epidermal cells **(a)** shown by very narrow angular distribution **(b)**. **(c,d)** Mixed orientation of cortical microtubules in *ktn1-2* petiole epidermal cells **(c)** and its quantitative demonstration **(d)**. **(e,f)** Cortical microtubule organization in the cotyledon of Col-0 **(e)** showing a single branching event (inset; arrow) and *ktn1-2* **(f)** showing multiple *de novo* branched initiation and formation of microtubules (**f**; arrows; inset). Scale bars: 10μm.

Petiole epidermal cells of Col-0 are elongated and as such they favor the predominant transverse orientation of cortical microtubules (Figures 1a,b). KATANIN 1 depletion in the *ktn1-2* mutant, promoted the isotropic growth of petiole epidermal cells and prevented the transverse orientation of cortical microtubules which rather occupied the cell cortex at random orientations (Figures 1c,d). Further inspection of cotyledon epidermal cells, showed that by comparison to Col-0 where microtubules where randomly oriented (Figure 1e), *ktn1-2* mutants exhibited similar patterns of organization. The only difference in this case was the observation of multiple branched nucleation events (Figure 1f, inset) compared to Col-0 where branching was more sparse (Figure 1e, inset).

**Figure 2:**
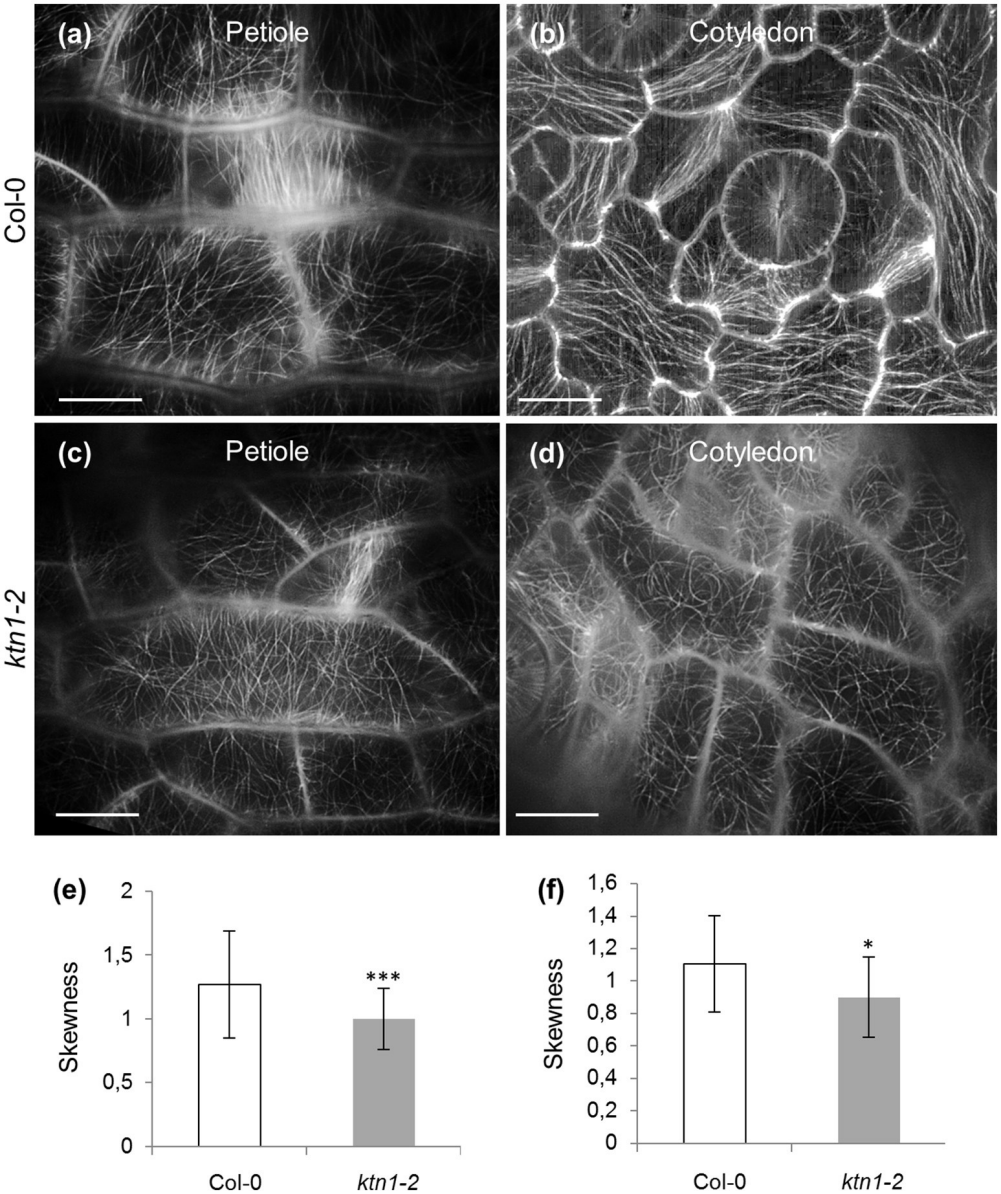
**Microtubule skewness in Col-0 and ***ktn1-2*** petiole and cotyledon epidermal cells expressing a GFP-TUA6 microtubule marker after SIM 2D imaging. (a–d)** Examples of microtubule organization in petiole **(a,c)** and cotyledon **(b,d)** epidermal cells of Col-0 **(a,b)** and *ktn1-2* **(c,d)**. **(e)** Skewness of petiole epidermal cells of Col-0 and *ktn1-2* (^***^p < 0.001; *N* = 49 cells for Col-0 and *N* = 34 for *ktn1-2*). **(f)** Skewness of cotyledon epidermal cells of Col-0 and *ktn1-2* (^*^p < 0.05; *N* = 28 cells for Col-0 and *N* = 26 for *ktn1-2*). Scale bars: 10 μm.

KATANIN 1 depletion also affected the degree of microtubule bundling in petiole and cotyledon epidermal cells. The above measure was quantified in terms of skewness (Figure 2). In the first case, it was found that bundling of cortical microtubules was significantly reduced in both petiole and cotyledon epidermal cells of *ktn1-2* (Figures 2c–f) as compared to Col-0 (Figures 2a,b,e,f).

**Figure 3:**
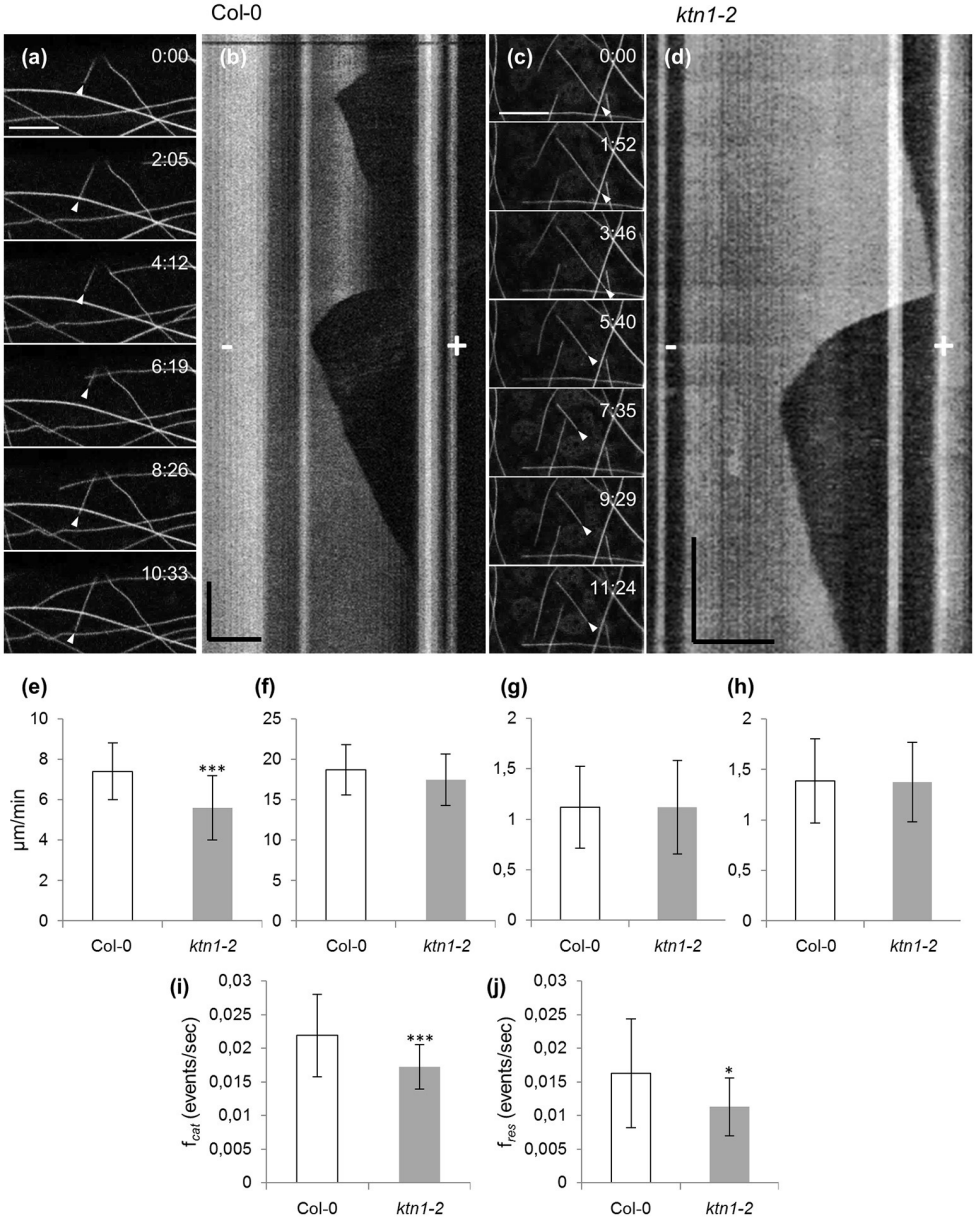
**Time lapsed 2D imaging of cotyledon epidermal cell cortical microtubules by CLSM and quantitative analysis of end-wise dynamics in Col-0 and ***ktn1-2*** mutant, stably expressing a GFP-TUA6 microtubule marker. (a,b)** A single microtubule from Col-0 exhibiting dynamic instability from the plus end (**a**, arrowhead) and the respective kymograph **(b)** with annotations of plus (+) and minus (−) ends. **(c,d)** An individual microtubule (**c**, arrowhead) from *ktn1-2* showing dynamic instability and the respective kymograph **(d)** with annotations of plus (+) and minus (−) ends. **(e–h)** Quantification of average values (± S.D) of plus end growth (**e**, *n* = 40 and 36 for Col-0 and *ktn1-2*, respectively; ^***^p < 0.001), shrinkage (**f**, *n* = 39 and 40 for Col-0 and *ktn1-2*, respectively), and minus end growth (**g**, *n* = 18 and 14 for Col-0 and *ktn1-2*, respectively) and shrinkage (**h**, *n* = 26 and 25 for Col-0 and *ktn1-2*, respectively). **(i,j)** Catastrophe (**i**, ^***^p < 0.001) and rescue (**j**, ^*^p < 0.05) frequencies (mean ± S.D) comparing Col-0 and *ktn1-2* (*n* = 39 microtubules for Col-0 and *n* = 40 microtubules *for ktn1-2*). These and other microtubule dynamics parameters can be found in Table 1. Scale bars: 10 μm **(a–c)** 5 μm **(d)**; Time bars: 1 min **(b)**, 2 min **(d)**. Numbers in **(a,c)** correspond to time (min:s).

Documentation of microtubule plus and minus end dynamics showed that by comparison to Col-0 (7.41 ± 1.42 μm/min; *N* = 40 microtubules from 12 cells; Figures 3a,b,e; Table 1), cortical microtubules of *ktn1-2* mutant show significantly slower plus end growth kinetics (5.6 ± 1.59 μm/min; *N* = 36 microtubules from 14 cells; *p* < 0.001; Figures 3c–e; Table 1). Other measures of microtubule dynamics, including plus end shrinkage (18.7 ± 3.14 μm/min for Col-0; *N* = 39 microtubules from 12 cells vs. 17.47 ± 3.19 μm/min for *ktn1-2*; *N* = 40 microtubules from 14 cells; Figure 3f; Table 1), minus end growth (1.12 ± 0.4 μm/min for Col-0; *N* = 18 microtubules from 12 cells vs. 1.12 ± 0.47 μm/min for *ktn1-2*; *N* = 14 microtubules from 8 cells; Figure 3g; Table 1), and minus end shrinkage (1.39 ± 0.42 μm/min for Col-0; *N* = 26 microtubules from 20 cells vs. 1.37 ± 0.4 μm/min for *ktn1-2*; *N* = 25 microtubules from 22 cells; Figure 3h; Table 1) revealed no significant differences. It was also noteworthy that *ktn1-2* mutants showed significantly lower catastrophe and rescue frequencies (0.017 ± 0.003 and 0.011 ± 0.004 events/s, respectively from a total of 40 microtubules compared to Col-0, 0.022 ± 0.006 and 0.016 ± 0.008 events/s, respectively from a total of 39 microtubules; Figures 3i,j; Table 1).

**Table 1:** **Features of microtubule dynamics in Col-0 and ***ktn1-2*** mutant**.

**Feature**	**Col-0**	***ktn1-2***	**Statistical significance of difference**
**MICROTUBULE DYNAMICS**			
+ end growth rate (mean ± SD; μm/min)	7.41 ± 1.42	5.6 ± 1.59	Significant *p* < 0.001
+ end shrinkage rate (mean ± SD; μm/min)	18.7 ± 3.14	17.47 ± 3.19	Not significant *p* = 0.08761
− end growth rate (mean ± SD; μm/min)	1.12 ± 0.4	1.12 ± 0.47	Not significant *p* = 0.994592
− end shrinkage rate (mean ± SD; μm/min)	1.39 ± 0.42	1.37 ± 0.4	Not significant *p* = 0.645752
Catastrophe frequency (mean ± SD; events × s^−1^)	0.022 ± 0.006	0.017 ± 0.003	Significant *p* < 0.001
Rescue frequency (mean ± SD; events × s^−1^)	0.016 ± 0.008	0.011 ± 0.004	Significant *p* < 0.05
Severing frequency (mean ± SD; events × 10^−3^ × μm^−2^ × s^−1^)	0.145 ± 0.0918	0	Significant *p* < 0.001

**Figure 4:**
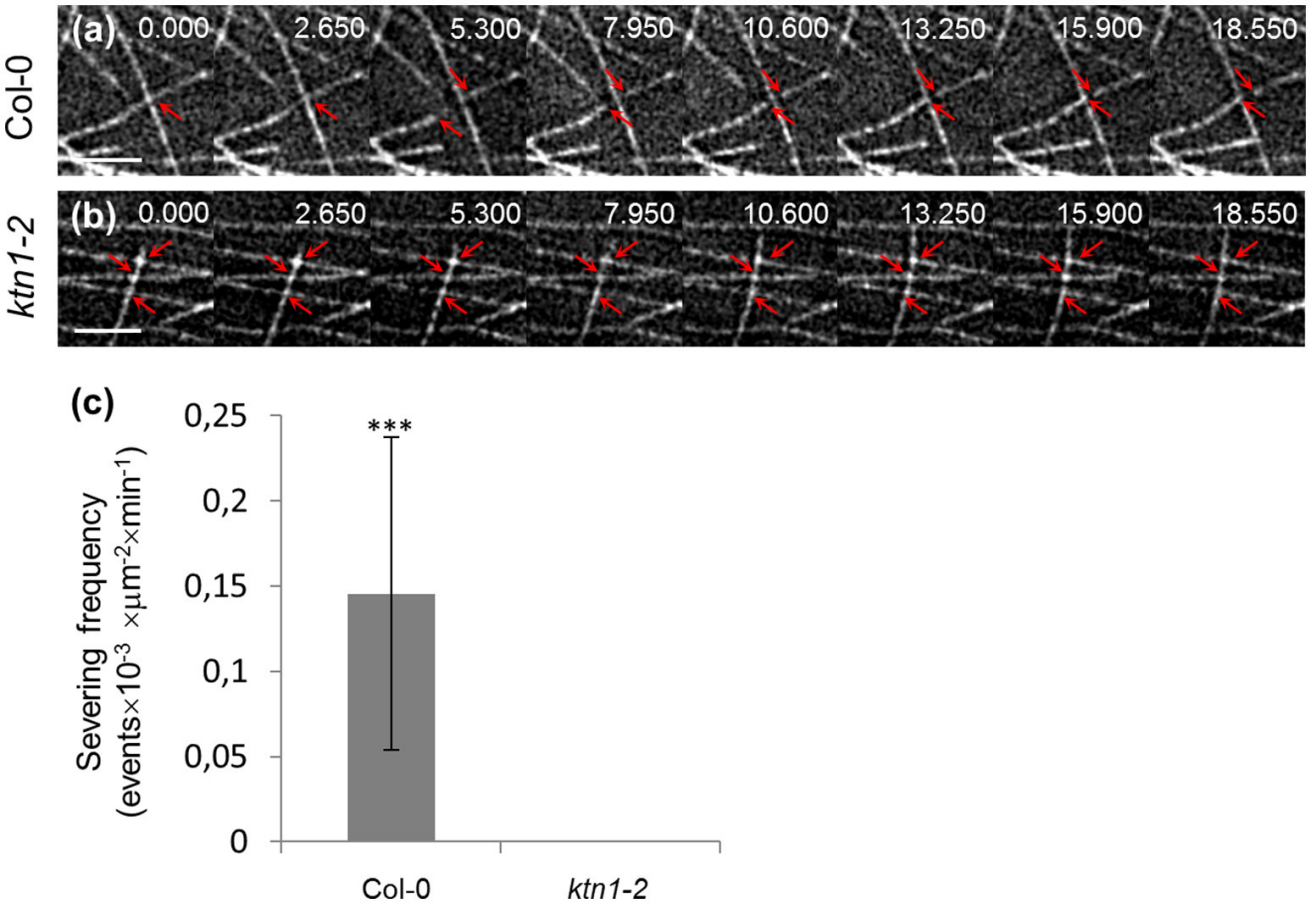
**Recording of severing events in Col-0 and ***ktn1-2*** petiole epidermal cells expressing a GFP-TUA6 microtubule marker with time-lapsed 2D SIM**. The spotted appearance of microtubules owes to the inhomogeneous incorporation of TUA6-GFP in the microtubule lattice (**a,b**; see Section Discussion by Komis et al., 2014). Time lapsed stills of petiole epidermal cell region showing severing in Col-0 **(a)** and absence of severing in *ktn1-2* **(b)**. **(c)** Quantitative comparison of severing frequencies in petiole epidermal cells of Col-0 and *ktn1-2* (^***^p < 0.001; *N* = 19 cells from Col-0 and *N* = 16 cells from *ktn1-2*). Severing frequencies can be found in Table 1. Arrows in **(a)** show microtubule crossover in first 2 frames and the microtubule ends resulting from severing in the rest of the frames. In **(b)** arrows in first frame show 3 microtubule crossovers where no severing is observed. Scale bars: 2 μm. Numbers at **(a,b)** correspond to time (s.ms).

As expected, *ktn1-2* mutants exhibit completely abolished severing activity resulting in the absence of severing in expected sites of the cortical array such as microtubule crossovers (Figures 4a–c; Table 1). In particular, the severing frequency in Col-0 petiole epidermal cells was 0.145 ± 0.0918 events × 10^−3^ × μm^−2^ (*N* = 19 cells) while in *ktn1-2* petiole epidermal cells the respective frequency was 0 (*N* = 16 cells). The absence of severing in *ktn1-2* mutants was most striking at predominant severing sites such as microtubule crossovers (e.g., Figure 4b).

### Defects in PPB formation and delayed PPB maturation

**Figure 5:**
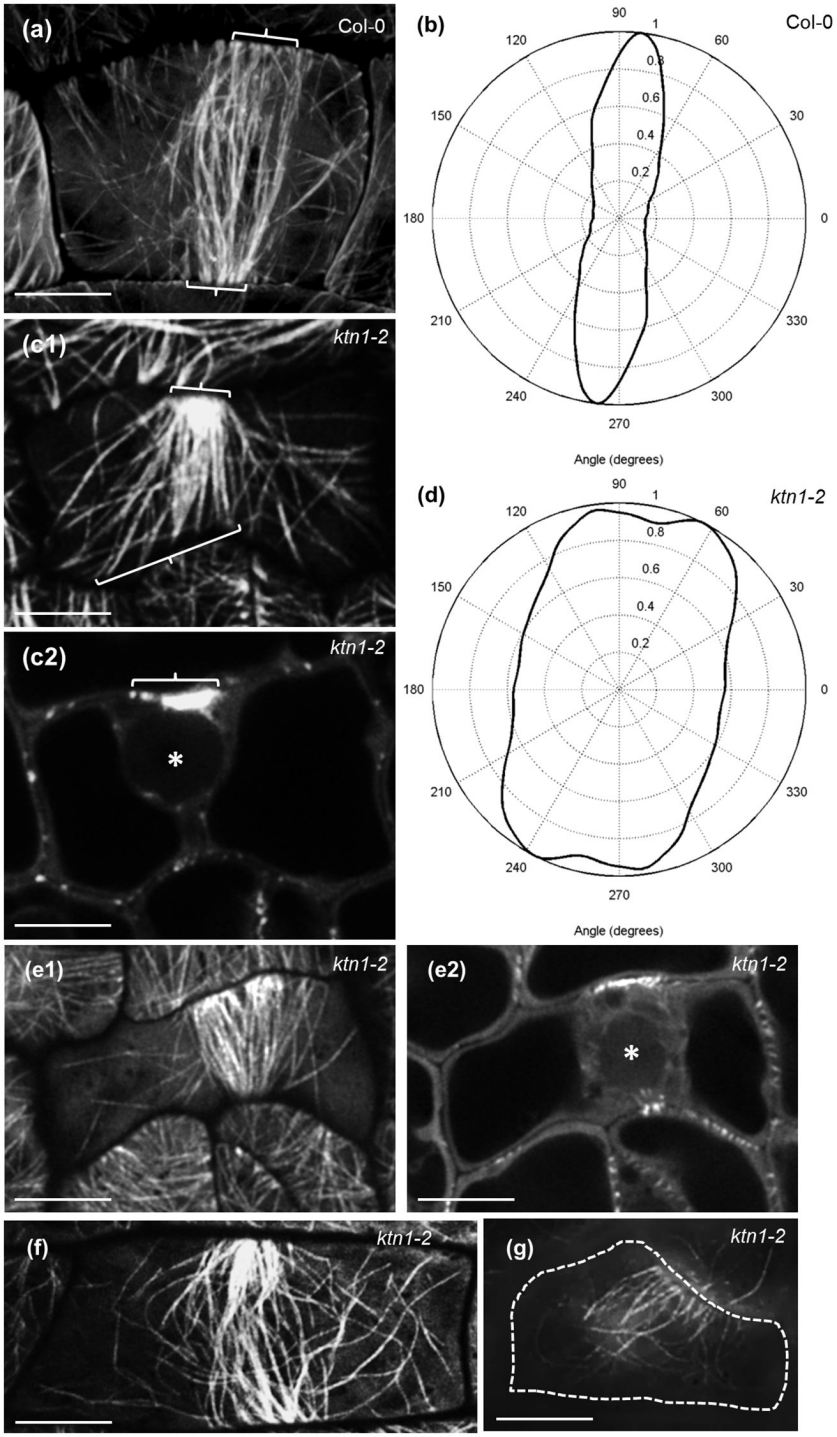
**PPB organization and MT anisotropy in Col-0 and ***ktn1-2*** mutants expressing a GFP-TUA6 microtubule marker**. Single optical sections were acquired with Airyscan CLSM **(a,c,e,f)** and SIM **(g). (a)** Typical early PPB (brackets) of Col-0 dividing petiole epidermal cell showing roughly parallel and homogeneous distribution of microtubules. **(b)** Angular distribution of microtubules in PPB of **(a)** showing a rather uniform transverse orientation. **(c1,c2)** Early PPB of *ktn1-2* petiole epidermal cell at surface **(c1)** and mid **(c2)** planes, showing a well-focused side and a much broader microtubule distribution on opposite side (**c1,c2** brackets) with long microtubules emanating from the PPB to the rest of the cortex. Scanning time was 4.28 min for the entire Z-stack while a rotating panoramic view can be found in Video S1. **(d)** Angular distribution of microtubules in PPB of **(c1)** showing much broader orientation than in **(a)** although with a transverse orientation trend. **(e–g)** More examples of abnormal PPB organization in *ktn1-2* including a fan-shaped PPB at surface **(e1)** and at middle (**e2;** asterisk denotes position of nucleus optical planes), a broad, disordered **(f)** and an incomplete one **(g)**. Asterisk in **(c2)** and **(e2)** indicates position of nucleus. Scanning time was 3.74 min for **(e1,e2)** and 17.8 s for **(f)**; **(g)** is a single optical section Scale bars: 5 μm.

Major defects in microtubule organization of *ktn1-2* mutant were observed in the case of PPB formation and narrowing as studied comparatively in Col-0 and *ktn1-2* seedlings stably transformed with a GFP-TUA6 microtubule marker. Col-0 cells exhibit a well-organized PPB comprising of parallel and straight microtubules (Figure 5a) with perpendicular orientation (Figure 5b). By contrast, *ktn1-2* preprophase cells mostly show very broad and often splayed PPBs with asymmetric organization containing over elongated and frequently bent microtubules localized outside of the PPB region (Figures 5c1,c2,d; Video S1) as well as fan-shaped, broad, disordered, and an incomplete PPBs (Figures 5e–g). It is also worth to note that many such PPBs of the *ktn1-2* mutant were incomplete as deemed by 3D rendering, failing to circumvent the entire cell cortex.

**Figure 6:**
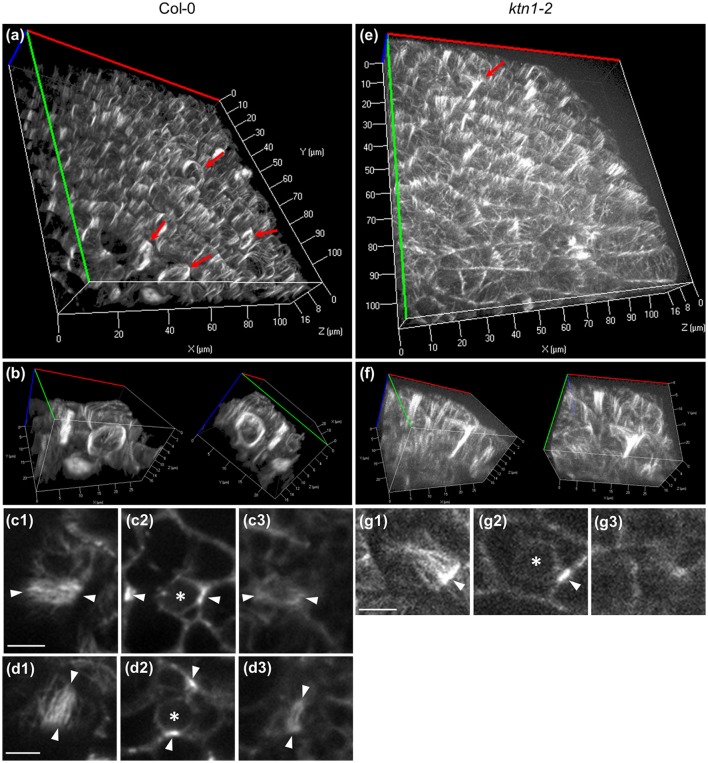
**Completeness of Col-0 and ***ktn1-2*** PPB in the three dimensions as visualized in cotyledon epidermal cells expressing a GFP-TUA6 microtubule marker after Z-optical sectioning and 3D reconstruction (a,b,e,f)** or after single optical section imaging **(c,d,g)** using spinning disc microscopy. **(a,b)** Overview **(a)** and higher magnification **(b)** of Col-0 cotyledon epidermis showing two adjacent cells with well-organized and complete PPBs at two different rotation angles. These cells are shown in a rotating panoramic view at Video S2. **(c,d)** Top **(c1,d1)**, middle **(c2,d2)**, and bottom **(c3,d3)** views of the PPBs shown in **(b)**. **(e,f)** Overview **(e)** and detail **(f)** of aberrant PPB formation in *ktn1-2* mutant at two different rotation angles. The same cell is shown in a rotating panoramic view at Video S3. **(g)** Top **(g1)**, middle **(g2)**, and bottom **(g3)** views of the *ktn1-2* PPB shown in **(f)**. Red arrows in **(a,e)** denote PPBs. Asterisks in **(c2,d2,g2)** denote nuclear position. Arrowheads in **(c,d,g)** denote the PPBs. For all Z-acquisitions shown herein, total scanning time was between 45 s **(a–d3)** to 75 s **(e–g3)**. Scale bars: 5 μm.

In the 3 dimensions, the PPB of Col-0 (Figures 6a–d3; Video S2) encompasses the entire cortical circumference with a rather uniform microtubule band. By contrast, PPBs of *ktn1-2* are non-uniform in width while sometimes they are incomplete failing to form a complete ring (Figures 6e–g3; Video S3). In quantitative terms, all PPBs examined in Col-0 (over 50 PPBs) were normal, while PPBs of *ktn1-2* were variably abnormal. In this case we discriminated 3 distinct categories: those with asymmetry of their organization extent (one side well organized and the other poorly organized; category I with 21 cases in total; Figures 5c1,c2,e1,e2), those which exhibited a roughly uniform width, however, with residual long cortical microtubules outside the PPB area (category II with 12 cases in total; Figure 5f) and those which were incomplete (category III with 8 cases in total; Figures 5g, 6g1–g3).

**Figure 7:**
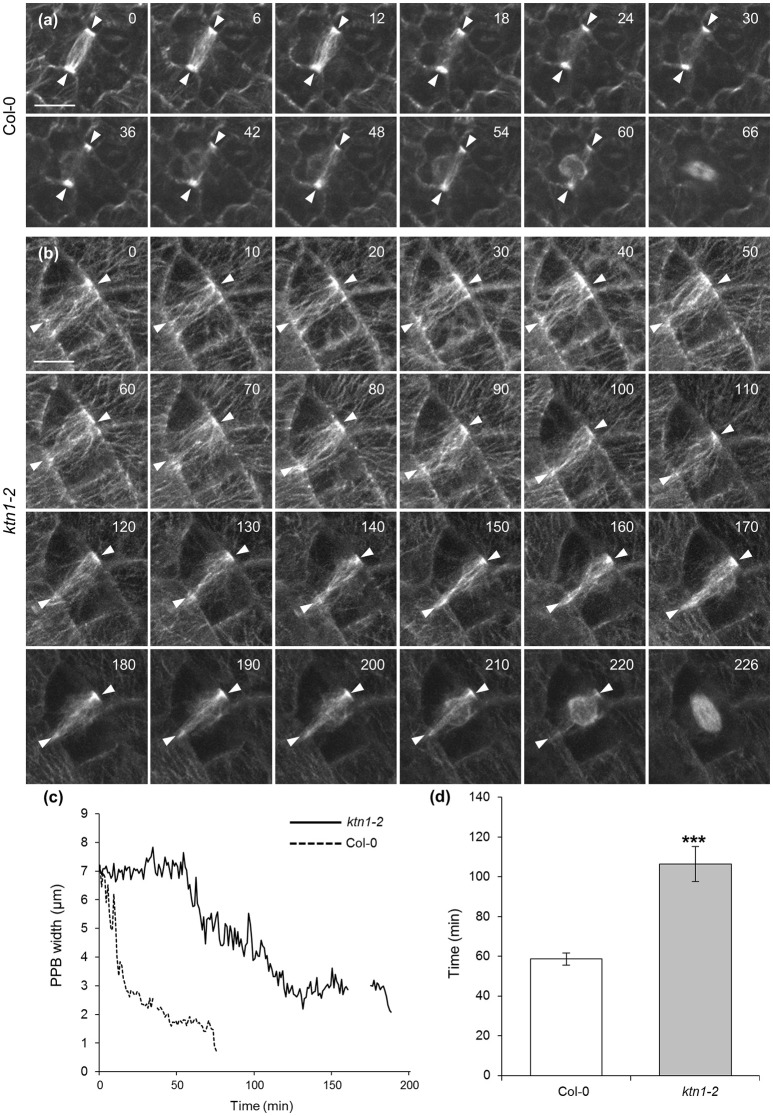
**Documentation and quantitation of PPB narrowing in dividing cells of Col-0 and ***ktn1-2*** mutant expressing a GFP-TUA6 microtubule marker after spinning disc time-lapse 2D imaging. (a)** Selected stills from the Video S4, showing the progressive narrowing of the PPB (arrowheads) in a Col-0 epidermal cell until entry in mitosis. **(b)** Selected stills from the Videos S5, S6 (continuation of mitotic progress of this cell is shown in Figure 9b), showing the progressive narrowing of the PPB (brackets) in a *ktn1-2* epidermal cell until entry in mitosis. **(c)** Line graph following the course of PPB narrowing by comparison between a Col-0 and a *ktn1-2* cell, showing significant delay in the latter. Interruptions in the lines correspond to interruptions in the recordings. **(d)** Averaged kinetic documentation of PPB narrowing (*N* = 6–7 cells from a total of 5 plants) showing significant delay of PPB narrowing in the *ktn1-2* mutant (^***^p < 0.001). Scale bars: 10 μm. Numbers in **(a,b)** correspond to time (min).

Time lapsed imaging and measurement of the PPB narrowing process showed that by contrast to PPB of Col-0 which narrows discernibly in a relatively short time (up to 1 h; Figures 7a,c,d; Video S4), the PPB of *ktn1-2* cells narrows at a much slower pace (sometimes exceeding 3 h) resulting in a broader PPB compared to Col-0 (Figures 7b–d; Video S5). Owing to the very low laser power used during documentation (0.07 mW at the focal plane of a 63 × 1.40 NA oil immersion objective) and the fact that such cells successfully enter and complete mitosis and cytokinesis (Videos S4, S6), phototoxicity can be excluded as the cause for the prolonged persistence of the PPB in *ktn1-2*. The time lapsed series, showed that sometimes, the different forms of PPB described above (Figure 5) maybe interchangeable to a certain extent. In this respect the cell shown in Figure 7b, Videos S5, S6, starts from category II (panels 0–90 min) and transforms to a reminiscent of category I (panels 100–210 min) although not as exaggerated as shown in Figures 5e1,e2.

### Progress of mitosis and cytokinesis

**Figure 8:**
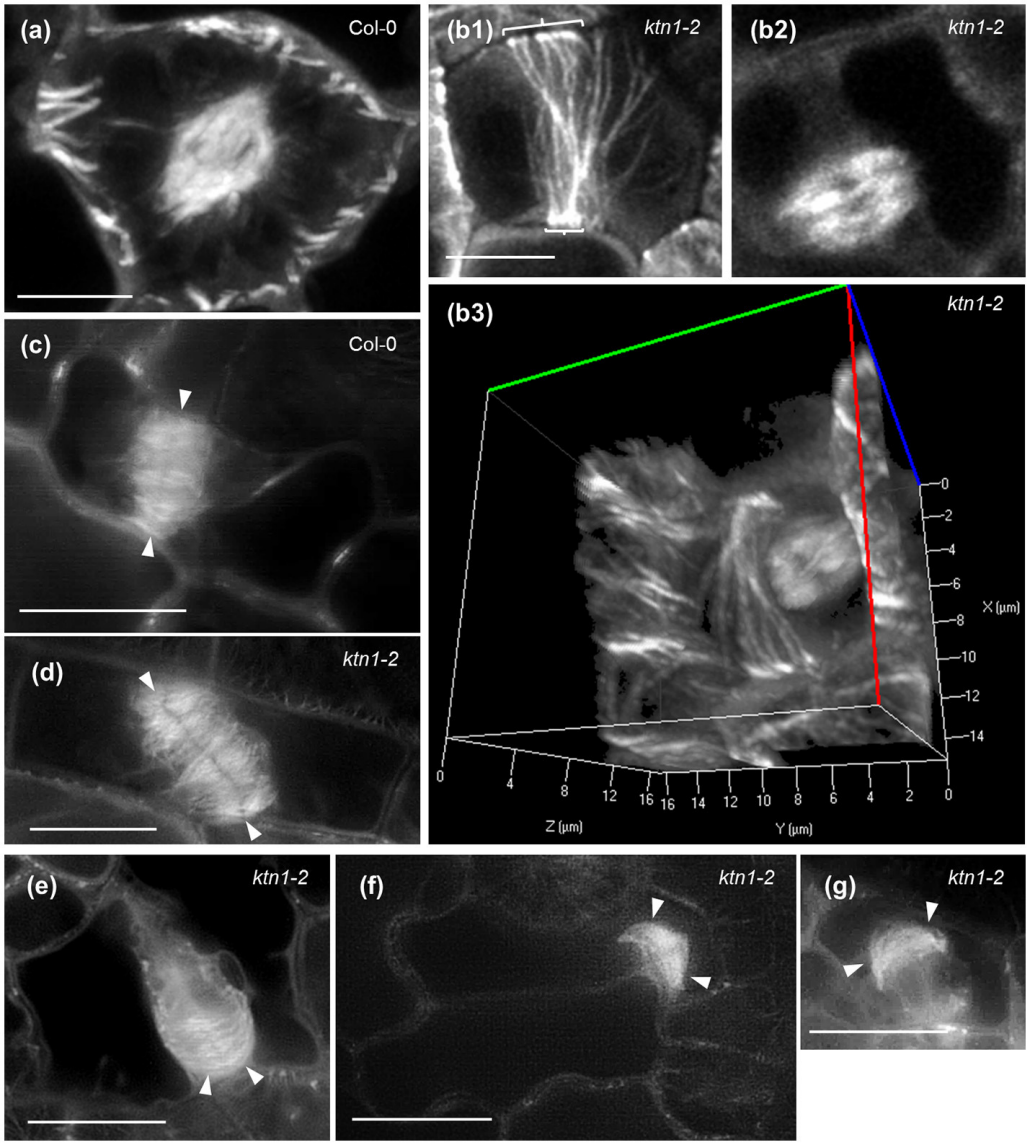
**Mitotic and cytokinetic forms in Col-0 and ***ktn1-2*** mutant expressing a GFP-TUA6 microtubule marker after Airyscan CLSM (a,b)** and SIM **(c–g)** imaging of single optical sections **(b1,b2,c–g)** or after 3D reconstruction **(a,b3). (a)** Typical prophase spindle in Col-0. **(b)** A typical bipolar metaphase spindle of *ktn1-2* which is formed in the presence of a broad PPB (brackets, **b1,b2**) at the cell cortex and a 3D rendered image of the same cell **(b3)** which corresponds to a panoramic 3D rotation of Video S7. Scanning time of the entire Z-stack (93 sections) was 5.9 min. **(c)** Typical phragmoplast of Col-0 (arrowheads). **(d)** An oblique phragmoplast of *ktn1-2* (arrowheads). **(e)** An advanced phragmoplast (arrowheads) of *ktn1-2* progressing asymmetrically toward the parent wall. **(f)** A similarly asymmetric phragmoplast of *ktn1-2* showing “arrowhead” configuration (arrowheads). **(g)** A shorter, advanced *ktn1-2* phragmoplast again with “arrowhead” configuration (arrowheads). All scale bars: 10 μm.

Mitotic spindle assembles normally in *ktn1-2* mutants as compared to Col-0 and assumes bipolarity quite early during prophase (Figures 8a,b). In all cases examined we never observed aberrations in mitotic spindle polarity as previously published (Panteris et al., 2011), however, we corroborated the residence of cortical microtubules reminiscent of PPB in late mitotic cells (Figures 8b1–b3; Video S7; see also Panteris et al., 2011). Likewise, the overall architecture of phragmoplast is conserved between Col-0 (Figure 8c) and *ktn1-2* mutants (Figures 8d–g), showing frequently the previously described “arrowhead” configuration, especially at late stages of phragmoplast expansion (Figures 8e–g).

**Figure 9:**
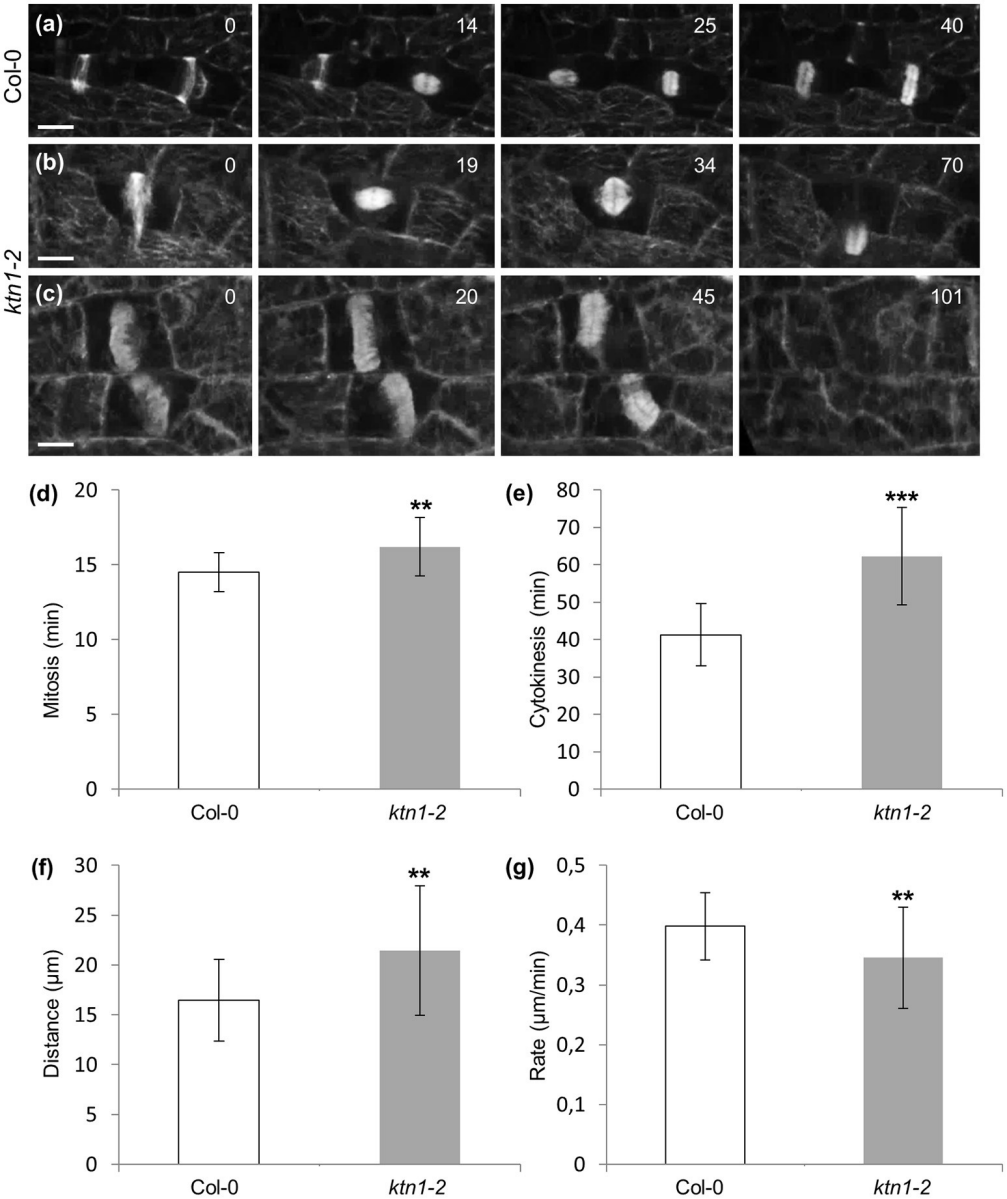
**Mitotic and cytokinetic progression in Col-0 and ***ktn1-2*** petiole epidermal cells expressing a GFP-TUA6 microtubule marker, after spinning disc 3D time-lapsed imaging. (a)** Transition from PPB to spindle and finally to phragmoplast formation and expansion in two dividing Col-0 petiole epidermal cells. Corresponds to Video S8. **(b)** Microtubule reorganization from PPB to spindle and to phragmoplast in a dividing *ktn1-2* petiole epidermal cell showing significant delay in phragmoplast expansion. Corresponds to Video S6 (showing continuation of mitotic progress in cell shown in Figure 7b). **(c)** Delayed phragmoplast expansion and bending in *ktn1-2*. Corresponds to Video S9. **(d–g)** Quantitative evaluation of mitotic progression comparing Col-0 and *ktn1-2*, showing duration of mitosis with significant delay of *ktn1-2* (**d**; ^**^p < 0.01; *N* = 20 for Col-0 and *N* = 45 for *ktn1-2*), cytokinesis (**e**; ^***^p < 0.001; *N* = 20 for Col-0 and *N* = 45 for *ktn1-2*), distance covered during expansion of phragmoplast (**f**; ^**^p < 0.01; *N* = 20 for Col-0 and *N* = 45 for *ktn1-2*) and rate of phragmoplast expansion (**g**; ^**^p < 0.01; *N* = 20 for Col-0 and *N* = 45 for *ktn1-2*). Scale Bars: **(a–c)** 10 μm, **(h–j)** 5 μm. Numbers in **(a,b)** correspond to time (min). Time required for an entire Z-stack was 8.6 s for **(a)**, 7.1 s for **(b)**, and 8.3 s for **(c)**.

Following the kinetics of the mitotic and cytokinetic processes and compared to Col-0 (Figures 9a,d; Video S8) mitotic progression is also slightly but significantly delayed in *ktn1-2* (Figures 9b,d; Video S6). In contrast to Col-0 (Figures 9a,e; Video S8) the course of centrifugal phragmoplast expansion is more appreciably delayed in *ktn1-2* (Figures 9b,c,e; Videos S6, S9). Although, the expanding phragmoplast of *ktn1-2* has to frequently span longer wall-to-wall distances compared to Col-0 (Figure 9f), it does so at considerably lower rates (Figure 9g). It is notable however, that albeit the prolongation of phragmoplast expansion, we never observed abortive cytokinesis and the phragmoplast always expanded until it met the parent wall (see next section).

### Positional relationships between PPB, mitotic spindle, and expanding phragmoplast

In Col-0 cells, mitotic spindle assembles in such way that its equatorial plane coincides with the plane set by the PPB. Previous studies have shown that at least in the case of the root, KATANIN 1 deficiency in either *erh3* or in *fra2* and *lue1* mutants (Webb et al., 2002; Panteris et al., 2011) results in disturbances of cell division plane orientation.

**Figure 10:**
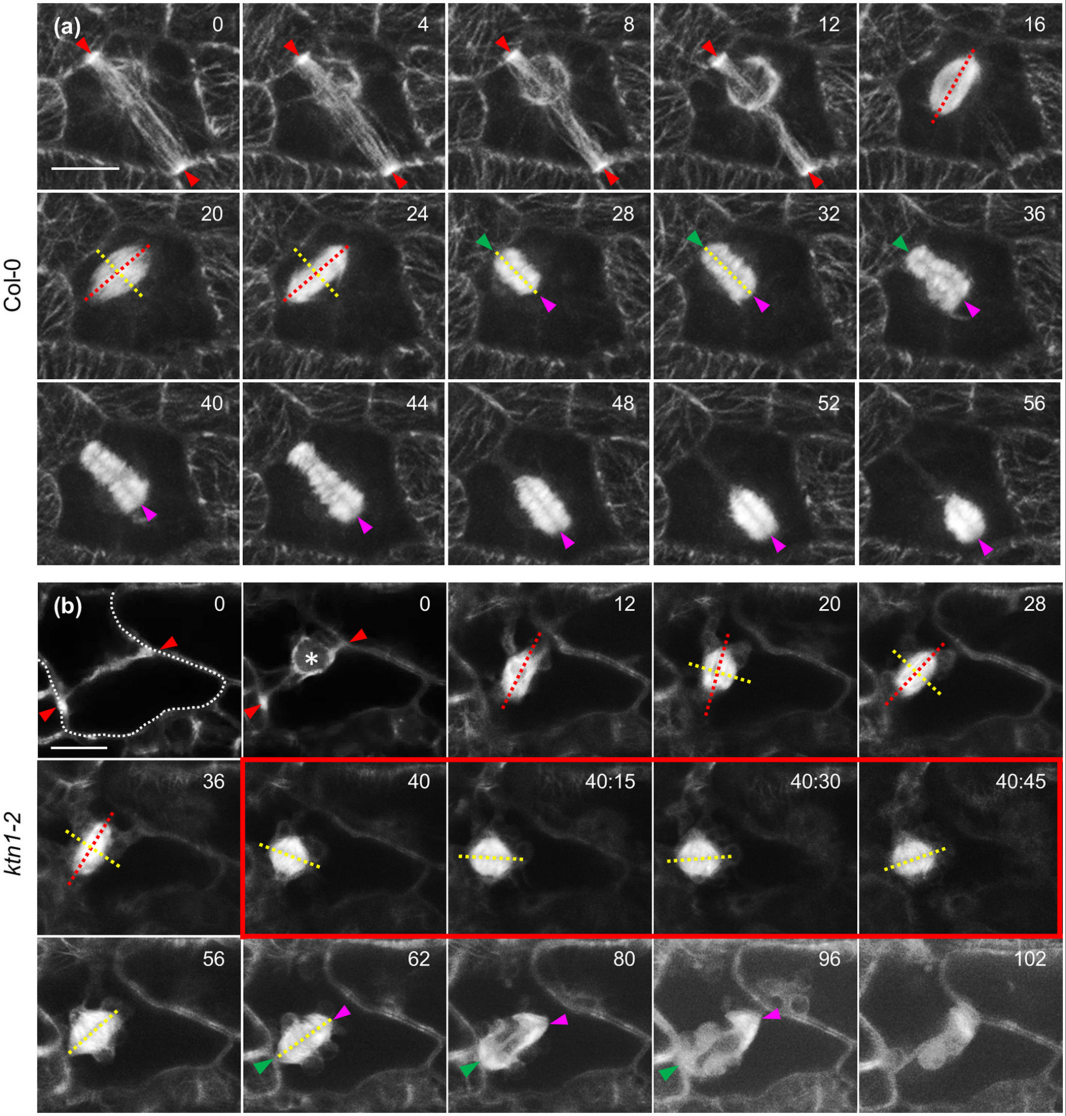
**Mitotic and cytokinetic progression in Col-0 and ***ktn1-2*** cotyledon epidermal cells expressing a GFP-TUA6 microtubule marker after Airyscan CLSM imaging. (a)** Mitotic spindle and phragmoplast dynamics in Col-0. Mitotic spindle is positioned so that the equatorial plane (yellow line) coincides with the PPB plane (red arrowheads) while the spindle axis (red line) is perpendicular to it. Both the spindle equator and the cell plate (yellow lines) remain coaligned to the CDP. Sometimes the phragmoplast reaches the parent wall asymmetrically creating a stable contact (green arrowhead) while the remaining leading edge (magenta arrowhead) still follows the trajectory predetermined by the PPB. **(b)** In *ktn1-2* the mitotic spindle equator (yellow line) is not adhering to the predetermined CDP (red arrowheads) and the spindle rotates significantly (spindle axis indicated by red line). Subsequently the nascent phragmoplast rotates as well until one end is attracted and tethered to the parent wall (green arrowhead). After this positional correction, the phragmoplast leading edge (magenta arrowhead) strictly follows the plane determined by the PPB so that the cell plate will coincide with the predetermined CDP. It is notable that progressively microtubules at the phragmoplast margin assume the “arrowhead” configuration as shown before (frames 80 and 96 min; Panteris et al., 2011). Scale Bars: 5 μm. Numbers in **(a,b)** correspond to time (min:s).

In dividing cells of Col-0, cell division plane is predetermined by the PPB. During mitosis the equatorial plane of the mitotic spindle coincides with the PPB plane and finally, it is followed by the expanding phragmoplast (Figure 10a). In dividing *ktn1-2* cells, the mitotic spindle assembles at a regular bipolar form (Figure 10b). However, it exhibits random rotational motions so that the equatorial plane deviates significantly from the PPB plane. After mitosis is completed and the phragmoplast is formed, it undergoes similar motions until one end meets the parent wall at the PPB site (Figure 10b, red outlined frames). As soon as one of the phragmoplast ends is stabilized in this manner the rest cytokinetic process is rectified so that the phragmoplast plane is expanding at the plane set by the PPB (Figure 10b, panels 80 and 96 min).

## Discussion

### Microtubule organization and dynamics in the *ktn1-2* mutant

Katanin is a master regulator of cortical microtubule organization (Nakamura, 2015) having essential roles in biasing their parallel arrangement during cell elongation. Previous studies showed that KATANIN 1 severs nascent microtubules formed by branched formation on preexisting microtubule walls (Nakamura et al., 2010) and becomes selectively activated at microtubule crossovers (Wightman and Turner, 2007; Wightman et al., 2013; Zhang et al., 2013). During the reorganization of cortical microtubules in response of blue light induction it was shown that a mechanism responsible for the selective activation of KATANIN 1 at microtubule intersections is via the activation of the PHOT1 and PHOT2 phototropin photoreceptors (Lindeboom et al., 2013). Additionally, KATANIN 1 was shown to regulate the mechanical response of cortical microtubule reorganization and establishment of anisotropy during the growth of the shoot apical meristem (Uyttewaal et al., 2012) by antagonizing the action of auxin (Sassi et al., 2014). Moreover, KATANIN 1 activity is regulating cytomorphogenesis of epidermal pavement cells, downstream of the small GTPase ROP6 (Lin et al., 2013).

In the present study, we quantified organizational defects of cortical microtubules in *ktn1-2* mutants when compared to Col-0. As parameters we used angular distribution which reflects to the ordering capacity of the cortical array, skewness which is a measure of microtubule bundling. Such correlative study was quite important since the severing activity of KATANIN 1 in the above cells is strikingly different, especially regarding severing at microtubule crossroads. In this case it seems, that such events are much more frequent in petiole than in cotyledon epidermal cells (Wightman et al., 2013).

**Figure 11:**
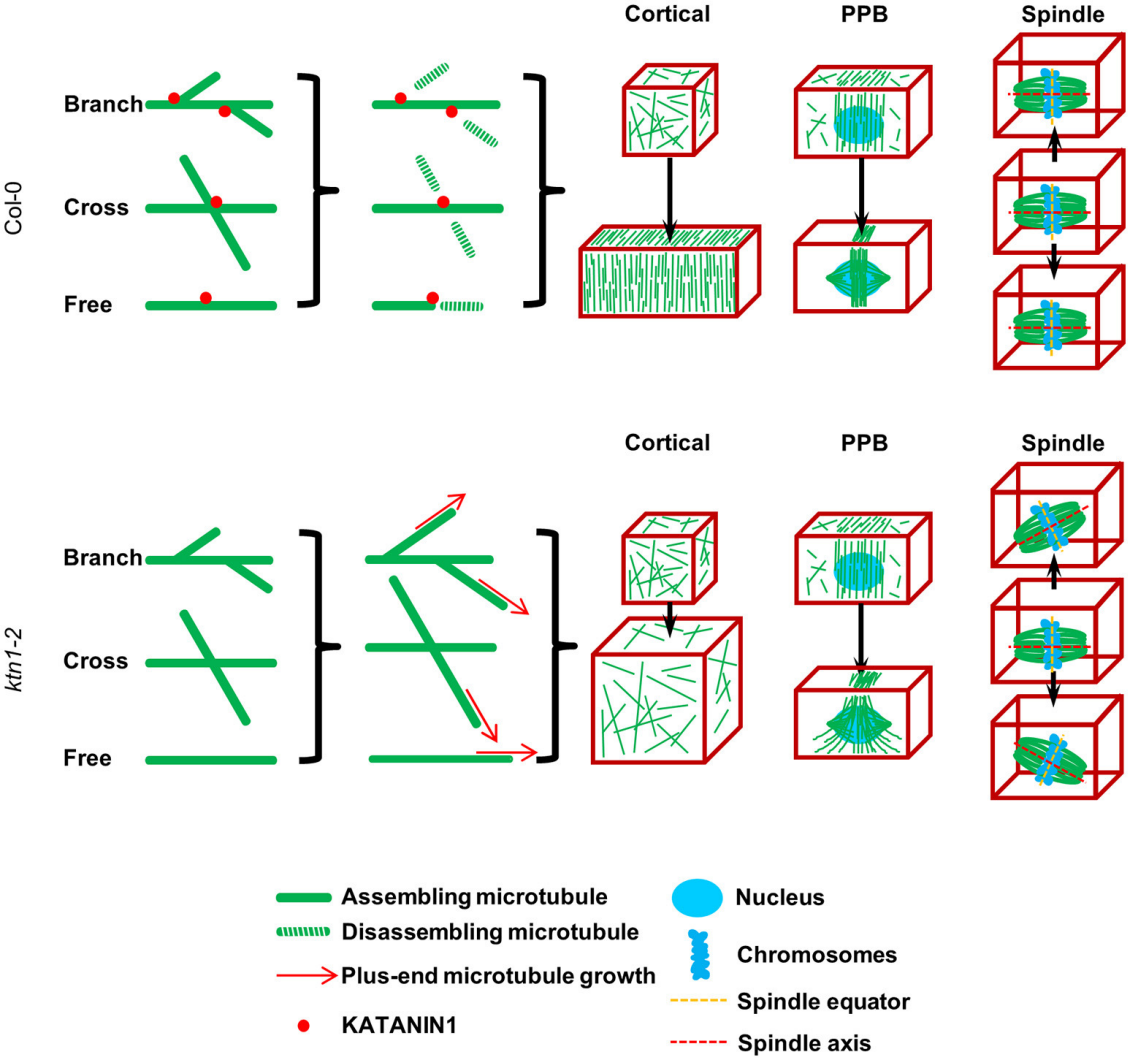
**A simplified schematic model explaining the defects of KATANIN 1 depletion in cell growth, PPB formation, and mitotic spindle positioning according to published and present data**. KATANIN 1 severs microtubules after branched formation (Nakamura et al., 2010), at microtubule crossovers (Wightman and Turner, 2007) apart from severing free microtubules. Through its activity, KATANIN 1 promotes the biased organization of microtubules at different stages of plant cell cycle. In interphase cells, KATANIN 1 microtubule severing induces parallel arrangement of cortical microtubules and supports unidirectional cell growth while in its absence as it occurs in *ktn1-2* mutant, cortical microtubule arrays remain disordered and result in isotropic cell growth. During PPB formation, KATANIN 1 severing activity promotes microtubule organization and confinement to the PPB zone while it is implicated in PPB narrowing. PPB formation and its course of maturation are impaired in *ktn1-2*. Finally, during mitosis, KATANIN 1 activity is related to mitotic spindle positioning by securing co-alignment of the equatorial plane with the CDP without affecting spindle formation, while in its absence the spindle exhibits considerable motions and appears to freely rotate in the cytoplasm. This model differs from that of Panteris et al. (2011) in that it appoints spindle rotation in KATANIN 1 mutants such as *ktn1-2* to a continuous dynamic process rather than attributing it to structural deficits caused during spindle formation (spindle multipolarity).

As schematically shown in Figure 11, the failure to sever microtubules at various situations depicted, can explain misorganization of cortical microtubules, since KATANIN 1 plays a key role to symmetry breaking and to anisotropy induction in the cortical array during polar or directed cell growth by biasing the parallel organization of cortical microtubules (Wightman and Turner, 2007, 2008; Nakamura et al., 2010; Uyttewaal et al., 2012; Lindeboom et al., 2013; Zhang et al., 2013; Chen et al., 2014; Sassi et al., 2014).

Within studies addressing the role of KATANIN 1 in cortical microtubules its impact on the dynamics of these microtubules was not addressed. In the present study we found that most measures of microtubule dynamics in *ktn1-2* cells were comparable to those of Col-0 with the exception of the plus-end growth rate (which was considerably reduced). Meanwhile the shrinkage rate was only slightly affected, but importantly the overall catastrophe frequency was also considerably reduced. As was shown by previous study on KATANIN 1 overexpression, it seems that KATANIN 1-mediated microtubule severing somehow favors microtubule bundling (Stoppin-Mellet et al., 2006). Therefore, the considerable reduction of microtubule bundling as judged by reduction in fluorescence skewness in *ktn1-2* petiole and cotyledon epidermal cells was expected. Microtubule bundling is considered as a major mechanism of ordered cortical microtubule organization mediated by members of the MAP65 protein family (Lucas et al., 2011; Lucas and Shaw, 2012). For this reason we propose that the effect of KATANIN 1 in cell growth directionality is not only direct by promoting the disassembly of microtubules at unfavorable orientations but also indirect by regulating microtubule bundling. These two discrete functions may explain cortical microtubule disordering and isotropic cell growth in the *ktn1-2* mutant. When katanin severs a microtubule, it promotes the exposure of GDP-tubulin ends which will inevitably undergo catastrophic shrinkage (Mace and Wang, 2015).

### Involvement of KATANIN in PPB formation and maturation and cell division plane determination

The present study is the first one to provide mechanistic insight on the involvement of KATANIN 1 in mitotic and cytokinetic progression of Arabidopsis cells through time lapsed imaging. The PPB is formed at the S to G2 interphase of the cell cycle by the large scale rearrangement of cortical microtubules to a broad annular microtubule assembly that progressively narrows until it finally disappears at the onset of mitosis concomitantly with nuclear envelope breakdown (Vos et al., 2004; Marcus et al., 2005; Azimzadeh et al., 2008). The duration of PPB persistence from its formation until its disappearance may be prolonged (e.g., over 11/2–2 h as recorded in tobacco BY-2 cells; Dhonukshe and Gadella, 2003; Vos et al., 2004) while in cases it may considerably exceed the duration of the entire mitotic procedure (as estimated for *Azolla pinnata*; Gunning et al., 1978). In our case, delays in PPB maturation/narrowing were inferred by comparison of the same cell types (i.e., dividing cotyledon pavement cells and dividing petiole epidermal cells). Of its known roles, the PPB accurately predicts the insertion of the succeeding cell plate during cytokinesis (Pickett-Heaps and Northcote, 1966; Rasmussen et al., 2011 and references therein) and represents a cortical site of intense endocytotic uptake (Karahara et al., 2009). Very interesting recent study proposed that PPB regulates robustness of cell division orientation by limiting spindle rotations (Schaefer et al., 2017). In this respect, we have found spatio-temporaly disturbed PPBs along with vigorous spindle and phragmoplast rotations in the *ktn1-2* mutant. They suggest KATANIN 1 role in the positioning of PPB, spindle, and phragmoplast.

Early steps of microtubule bundling during the onset of PPB formation are mediated by short actin microfilaments (Takeuchi et al., 2016) while the occurence of actin within the PPB is considered to be crucial for its narrowing which is prevented upon actin depolymerization (Granger and Cyr, 2001). The inability of *ktn1-2* preprophase cells to form a regular and progressively narrowing PPB, implicates KATANIN 1 microtubule severing activity in controlling microtubule assembly during both processes. Therefore, it is not surprising that ectopic microtubules, i.e., microtubules that reach cortical areas outside the PPB zone, seem to emanate from the PPB.

The role of PPB in the determination of cell division plane orientation has been extensively documented through the localization of cortical marker proteins such as TANGLED, FASS, PHRAGMOPLAST ORIENTING KINESINS 1 and 2, AIR9, and RanGAP1, the localization of which either coincides or even persists at the cortical PPB site throughout mitosis and cytokinesis (Camilleri et al., 2002; Buschmann et al., 2006; Müller et al., 2006; Walker et al., 2007; Xu et al., 2008). Beyond the localization of the above protein markers at the PPB site and the defects of cell division plane orientation observed in Arabidopsis mutants of the above proteins, the mechanisms attracting the phragmoplast at the cortical cell division plane and guiding cell plate deposition coincidentally to the PPB plane remain largely elusive.

In accordance to previous localization of KATANIN 1 within the PPB (Panteris et al., 2011), we postulate that KATANIN 1 is not responsible for clearing the rest of the cell cortex from residual microtubules but rather that KATANIN 1 activity is restricted within the PPB to regulate microtubule length and in this way KATANIN 1 depletion in *ktn1-2* perturbs PPB shape, organization and position. From our kinetic analysis, it became also evident that PPB narrowing is considerably prolonged, further suggesting that KATANIN 1 activity is also implicated in PPB maturation.

In such wide PPB areas defined in *ktn1-2* mutants, it is hypothetically possible that markers of CDP orientation attracting cell plate deposition to the PPB site (Lipka et al., 2015) may be more diffusely localized and this would help to explain the occurence of oblique cell division planes observed in KATANIN 1 mutants such as *erh3, fra2, lue1*, and *ktn1-2* (e.g., Webb et al., 2002; Panteris et al., 2011). In living mitotic petiole and cotyledon epidermal cells of *ktn1-2* we never observed multipolar spindles as those described before by tubulin immunofluorescence in fixed root whole mounts of *fra2* and *lue1* (Panteris et al., 2011). Mitotic spindles of *ktn1-2* were uniformly bipolar and identical in shape compared to those of Col-0. What differed remarkably between the two samples (Col-0 and *ktn1-2*) was that mitotic spindles of *ktn1-2* exhibited large scale rotational motions with the equatorial plane deviating considerably from the CDP as it is defined by the PPB. After the completion of mitosis, the young phragmoplast is also very motile showing a propensity to co-align with the PPB site. Once one part of the phragmoplast is stably anchored to the parent wall, it does so on the PPB site while the remaining of phragmoplast margin moves along the PPB plane until it meets the opposite PPB cortical site. By contrast to what was published before (Panteris et al., 2011) spindle polarity, form, or position, is not related to CDP orientation since cell plate deposition uniformly follows the PPB plane in *ktn1-2* irrespectively of spindle orientation. Therefore, whenever CDP orientation defects are observed in KATANIN 1 mutants (Webb et al., 2002; Panteris and Adamakis, 2012) they can be attributed to PPB malorganization or misorientation.

In *ktn1-2* mutant most of the PPBs documented show an asymmetry with respect to their organization degree with one very well focused and one poorly and very broad side. In this case, it might be that the expanding phragmoplast might establish firm contact with the cortical site corresponding to the well-organized PPB side, however, it would have problems to navigate toward the ill-defined side.

### Involvement of KATANIN 1 in progression of mitosis and cytokinesis

In dividing epidermal cells of the petiole or the cotyledon of *ktn1-2* mutant, the spindle forms normally prior to PPB breakdown. As a matter of fact, the mitotic spindle may progress to metaphase in the presence of prominent residual PPB-like cortical microtubule structure, suggesting that the complete disassembly of the PPB is not a prerequisite for normal mitotic spindle assembly.

The mitotic spindle of the *ktn1-2* mutant at any stage of its organization (prophase, metaphase, and anaphase) does not seem to differ morphologically from its Col-0 counterpart, further implying that KATANIN 1 microtubule severing is not required to guide spindle assembly and transitions. To this extend we never observed the previously reported spindle multipolarity during prophase as reported before (Panteris et al., 2011; Panteris and Adamakis, 2012). Moreover, and in contradiction to animal cells, *A. thaliana* KATANIN 1, does not seem to affect mitotic spindle size as was shown through comparison between two different Xenopous species (Loughlin et al., 2011).

In contrast to Col-0 where the spindle showed minimal movement in the cytoplasmic space, *ktn1-2* mitotic spindles at any stage displayed broad rotational motions such that the equatorial plane significantly deviated from the PPB plane. This observation suggests that KATANIN 1 activity is somehow required for spindle positional control, possibly by controlling attachment of the spindle to the cell cortex (Granger and Cyr, 2001; Kojo et al., 2013, 2014; Lipka et al., 2015). Similar motions were also exhibited by the nascent phragmoplast at the earliest stages of its formation and before its margin could become tethered to the parent wall. Nevertheless, the motions of the phragmoplast can be considered as corrective as they always concluded with the attachment of its margin to predetermined cortical sites. Therefore, anchoring of the phragmoplast to the “correct” cortical sites (i.e., those determined by the PPB) is not prevented in the *ktn1-2* mutant.

KATANIN 1 depletion also interferes with the course of mitosis but most remarkably with the duration of cytokinesis, although it does not prevent the complete cell plate deposition, neither does it uncouple its positional relationship with the PPB. The centrifugal expansion of the phragmoplast requires a continuous assembly of microtubules at its margin followed by microtubule disassembly at the lagging parts toward the center of the cell plate (Murata et al., 2013). Similar delays in phragmoplast expansion were observed in the Arabidopsis signaling mutant *mpk4*, lacking the activity of the mitogen activated protein kinase 4 (Beck et al., 2011). Such results indicate that the coordinated microtubule unbundling via MPK4-dependent phosphorylation of members of the MAP65 microtubule bundling proteins (Beck et al., 2010; Sasabe et al., 2011) and KATANIN 1-mediated microtubule severing, may facilitate the clearing of microtubules from the lagging areas of the phragmoplast and allow its centrifugal expansion solely at the margin.

## Author contributions

GK and MO conducted all live cell imaging. GK and IL made all post acquisition analyses with input from MO, DS, and IL generated material pertinent to this study. OŠ helped with quantitative evaluations. GK wrote first draft of the manuscript with input from all co-authors. JŠ conceived and supervised study, helped to interpret data and finalized manuscript.

### Conflict of interest statement

The authors declare that the research was conducted in the absence of any commercial or financial relationships that could be construed as a potential conflict of interest.

## Supplementary material

Video S1 **Panoramic 3D rendering of the cell shown in Figure 5c**.

Video S2 **Panoramic 3D rendering of the cell shown in Figure 6b**.

Video S3 **Panoramic 3D rendering of the cell shown in Figure 6f**.

Video S4 **Time lapsed series of the cell shown in Figure 7a**.

Video S5 **Time lapsed series of the cell shown in Figure 7b**.

Video S6 **Time lapsed series of the cell shown in Figure 9b (Video starts from the end of Video S5)**.

Video S7 **Panoramic 3D rendering of the cell shown in Figure 8b**.

Video S8 **Time lapsed series of the cell shown in Figure 9a**.

Video S9 **Time lapsed series of the cell shown in Figure 9c**.
